# Steroids Enable Mesenchymal Stromal Cells to Promote CD8^+^ T Cell Proliferation Via VEGF‐C

**DOI:** 10.1002/advs.202003712

**Published:** 2021-05-02

**Authors:** Yurun Gan, Tao Zhang, Xiaodong Chen, Wei Cao, Liangyu Lin, Liming Du, Yu Wang, Fei Zhou, Xuefeng He, Yulong He, Jianhe Gan, Huiming Sheng, Lydia Sorokin, Yufang Shi, Ying Wang

**Affiliations:** ^1^ CAS Key Laboratory of Tissue Microenvironment and Tumor Shanghai Institute of Nutrition and Health University of Chinese Academy of Sciences Chinese Academy of Sciences Shanghai 200031 China; ^2^ Cyrus Tang Hematology Center Collaborative Innovation Center of Hematology State Key Laboratory of Radiation Medicine and Protection Cam‐Su Genomic Resources Center Soochow University Suzhou China; ^3^ The Third Affiliated Hospital of Soochow University The First Affiliated Hospital of Soochow University State Key Laboratory of Radiation Medicine and Protection Institutes for Translational Medicine Soochow University Medical College Suzhou Jiangsu 215123 China; ^4^ Tongren Hospital Shanghai Jiao Tong University School of Medicine Shanghai 200336 China; ^5^ Institute of Physiological Chemistry and Pathobiochemistry University of Muenster Muenster 48149 Germany

**Keywords:** CD8^+^ T cells, graft‐versus‐host disease, mesenchymal stromal cells, steroids, vascular endothelial growth factor C, VEGFR3

## Abstract

Mesenchymal stromal cells (MSCs) function as a formidable regulator of inflammation and tissue homeostasis and expanded MSCs are shown to be effective in treating various inflammatory diseases. Their therapeutic effects require the existence of certain inflammatory cytokines. However, in the absence of sufficient proinflammatory stimuli or in the presence of anti‐inflammatory medications, MSCs are animated to promote immune responses and unable to alleviate inflammatory disorders. In this study, it is demonstrated that steroid co‐administration interferes the efficacy of MSCs in treating acute graft‐versus‐host disease (aGvHD). Molecular analysis reveals that vascular endothelial growth factor C (VEGF‐C) is highly induced in MSCs by steroids and TNF*α* and VEGF‐C in turn promotes CD8^+^ T cell response. This immune promoting effect is abolished by blockade or specific genetic ablation of VEGFR3 in CD8^+^ T cells. Additionally, administration of VEGF‐C alone exacerbates aGvHD progression through eliciting more vigorous CD8^+^ T cell activation and proliferation. Further studies demonstrate that VEGF‐C augments the PI3K/AKT signaling process and the expression of downstream genes, such as Cyclin D1. Thus, the data demonstrate that steroids can reverse the immunosuppressive effect of MSCs via promoting VEGF‐C‐augmented CD8^+^ T cell response and provide novel information for designing efficacious MSC‐based therapies.

## Graphical abstract

Steroids are first‐line drugs for treating acute graft‐versus‐host disease (aGvHD). Recently, mesenchymal stromal cells (MSCs) have emerged as a superb choice. However, concomitant use of both worsens the disease course. This manuscript reveals that steroids, together with TNF*α*, synergistically induce MSCs to produce abundant vascular endothelial growth factor C, which enhances CD8^+^ T cell response and exacerbates aGvHD progression.

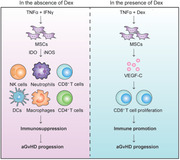

## 1 Introduction

The cardinal traits of mesenchymal stromal cells (MSCs) during tissue repair and regeneration are their concerted actions of immunoregulation, production of multiple growth factors, and multiple differentiation potential under certain conditions.^[^ ^1^ ^]^ The discovery of the prominent immunosuppressive properties of MSCs in vitro raises the possibility for their application in the treatment of autoimmune diseases.^[^ ^2^ ^]^ Indeed, the first case report that exogenously administered MSCs could successfully alleviate refractory acute graft‐versus‐host disease (aGvHD) leading to extensive investigations on the applications of MSCs in various autoimmune diseases.^[^ ^3^ ^]^ Many studies have demonstrated that the therapeutic effects of MSCs on autoimmune diseases variably rely on their high expression of immunosuppressive factors, including indoleamine 2,3‐dioxygenase (IDO),^[^ ^4^ ^]^ inducible nitric oxide synthase (iNOS),^[^ ^5^ ^]^ transforming growth factor‐*β*(TGF‐β),^[^ ^6^ ^]^ insulin like growth factor 2,^[^ ^7^ ^]^ hepatic growth factor,^[^ ^8^ ^]^ TNF‐stimulated gene 6,^[^ ^9^ ^]^ and prostaglandin 2,^[^ ^10^ ^]^ which endow MSCs the capabilities of suppressing proinflammatory responses or switching proinflammation to anti‐inflammation.

The immunosuppressive property of MSCs is not intrinsic, rather activated by certain combinations of inflammatory cytokines, IFN‐*γ* with TNF*α*, or IL‐1*β*.^[^ ^11^ ^]^ Blockade of IFN‐*γ* or employment of MSCs derived from IFN*γ*R1^−/−^ mice demonstrated that the activation of IFN*γ*R1 on MSCs is a prerequisite for the acquisition of the immunosuppressive capacity of MSCs.^[^ ^11^ ^]^ In response to the dynamic changes of inflammatory factors, the immunoregulation of MSCs is highly plastic.^[^ ^1^ ^]^ It has been reported that administration of MSCs could successfully inhibit aGvHD in the presence of vigorous inflammation such as that in steroid and cyclosporine A (CsA) resistant GvHD patients. However, their therapeutic effects cannot be observed when MSCs were infused before the initiation of inflammation, or on the day of bone marrow transplantation (BMT).^[^ ^12^ ^]^ In addition, low level of inflammation or presence of anti‐inflammatory medications, such dexamethasone (Dex), and CsA, abolishes the immunosuppressive capacity of MSCs, and interferes with the efficacy of MSC‐based treatment of liver fibrosis^[^ ^13^ ^]^ and delayed type hypersensitivity.^[^ ^14^ ^]^

Most insights into the immunoregulatory properties of MSCs came from the exploration of their therapeutic effects on inflammatory diseases. Administration of MSCs exhibited astonishing efficacy in treating a 9‐year‐old patient suffering from CsA‐ and steroid‐resistant grade IV aGvHD.^[^ ^3^ ^]^ However, in a US Food and Drug Administration approved clinical trial, no significant improvement was observed when the data of a large scale MSC‐based aGvHD trial were analyzed.^[^ ^15^ ^]^ These conflicting results raise fundamental issues for how to successfully apply MSCs in patients suffering from various types and stages of inflammatory diseases. Particularly, we need to understand to what extent do MSCs exert their effects on immunosuppression and tissue repair under various pathological settings, and what are the underlying molecular mechanisms for the variation in the therapeutic effects.

Here, we reported that steroids could abolish the therapeutic effect of MSCs on aGvHD and eliminate their suppression on CD8^+^ T cells. In the presence of steroids, MSCs could enhance the proliferation CD8^+^ T cells. Among various paracrine factors produced by steroid‐treated MSCs, we found that vascular endothelial growth factor C (VEGF‐C) is highly produced and is responsible for the exacerbation of aGvHD progression. In vivo, mice with VEGFR3 conditional deletion in CD8^+^ T cells demonstrated that VEGFR3 signaling mediates the activation and proliferation of CD8^+^ T cells. More significantly, in aGvHD patients, high level of VEGF‐C is related to the condition of refractory aGvHD. Targeted inhibition of VEGFR3 signaling could improve the efficacy of MSCs in aGvHD, even concomitant with steroid administration. We believe that these findings have important ramifications for the understanding of MSC‐mediated immunoregulation and their efficacy in the treatment of autoimmune diseases.

## 2 Results

### 2.1 Steroids Alleviate MSC‐Mediated Therapeutic Effect on aGvHD

**Figure 1:**
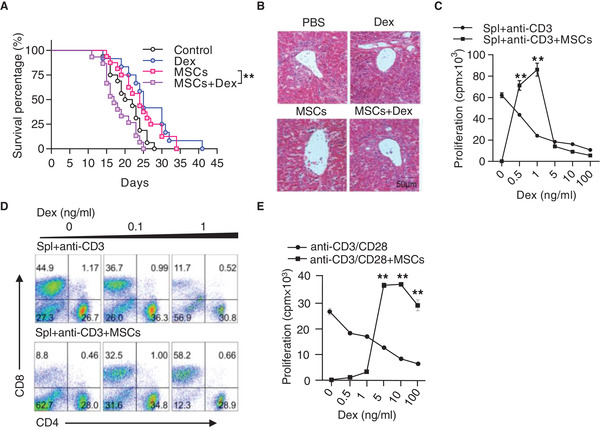
Steroids abolish the therapeutic effect of MSCs on aGvHD. A) Survival curves. Recipient mice (C57BL/6 × C3H F1) were lethally irradiated, followed by transplantation of bone marrow cells (5 × 10^6^) and splenocytes (5 × 10^7^) derived from C57BL/6 mice to induce aGvHD. Recipients were treated with MSCs (5 × 10^5^) on days 5 and 7 after bone marrow transplantation and with and without Dex (2 mg kg^−1^) treatment (3 times per week). Data were pooled from 3 independent experiments (Control: *n =* 16, Dex: *n =* 12, MSCs: *n =* 16, Dex + MSCs: *n =* 15). B) Hematoxilin & eosin staining of the liver sections on day 21 after bone marrow transplantation. Scale bar: 50 µm. C) Dex reversed MSC‐induced immunosuppression on splenocyte proliferation. Splenocytes (2 × 10^5^) were co‐cultured with MSCs (1 × 10^4^) in the presence of anti‐CD3, with Dex treatment at indicated concentrations for 48 h. Cell proliferation was measured by ^3^H‐thymidine incorporation. Spl: splenocytes. D) Flow cytometric analysis of the percentages of CD4^+^ T cells and CD8^+^ T cells in the co‐culture system of activated splenocytes and MSCs, with or without Dex. E) Dex reversed MSC‐induced immunosuppression on CD8^+^ T cell proliferation. Splenic CD8^+^ T cells were isolated from C57BL/6 mice and co‐cultured with MSCs in the presence of anti‐CD3 and anti‐CD28, with or without Dex. Cell proliferation was assayed by the last 6‐h ^3^H‐thymidine incorporation. Results are representative of 3 independent experiments. Data are presented as mean ± SEM. ^**^ *p* < 0.01.

To investigate the inefficacy of MSCs in aGvHD treatment, especially in patients with concomitant administration of steroids, we established a mouse aGvHD model by injecting nucleated bone marrow cells and splenocytes from C57BL/6 mice into lethally irradiated F1 (C57BL/6 × C3H) mice and assessed the effects of Dex, bone marrow MSCs (BM‐MSCs), or both on aGvHD. The BM‐MSCs were identified and expanded in vitro as specified in Figure S1A,B, Supporting Information. Consistent with previous studies, administration of Dex or MSCs alone significantly prolonged the survival of aGvHD mice, however, concomitant injection of Dex and MSCs did not show improvement and in fact worsened the disease and increased mortality (**Figure** **1A**). The severity was also confirmed by leukocyte infiltration in the liver (Figure 1B).

We then analyzed the interaction of Dex and MSCs in modulating immune responses. Isolated splenocytes cultured with MSCs were activated with anti‐CD3 in the presence of Dex. When the proliferation of suspensible activated T cells was assessed by tritiated thymidine incorporation, it was found that the presence of MSCs significantly inhibited the proliferation of T cells as previously shown.^[^ ^11^ ^]^ However, the addition of Dex to the activated splenocyte culture significantly diminished the inhibitory effect of MSCs on T cell proliferation (Figure 1C). This effect was also observed when the absolute numbers of splenocytes were enumerated (Figure S1C, Supporting Information). When different T cell subsets were analyzed, we found that the frequency of CD8^+^ T cells was markedly enhanced in activated splenocyte culture in presence of both MSCs and Dex, whereas the frequency of CD4^+^ T cells was not affected in the same culture (Figure 1D; Figure S1D,E, Supporting Information).

We then examined the effect of Dex on MSC‐mediated suppression of CD8^+^ and CD4^+^ T cells, respectively. Naïve CD8^+^ or CD4^+^ T cells were purified with specific antibody coated magnetic beads and co‐cultured with MSCs in the presence of anti‐CD3 and anti‐CD28, with or without Dex. Interestingly, we found that only purified CD8^+^ T cells showed significant enhancement of proliferation in the presence of both Dex and MSCs (Figure 1E). No significant change in CD4^+^ T cell proliferation was observed (Figure S1F, Supporting Information). Thus, the abolishment of the efficacy of MSCs on aGvHD by Dex is through promoting CD8^+^ T cell response.

### 2.2 VEGF‐C Mediates Steroid and MSC‐Promoted CD8^+^ T Cell Response

**Figure 2:**
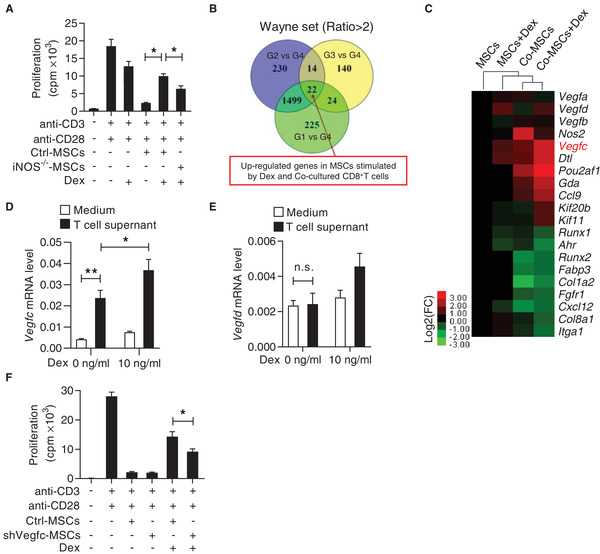
VEGF‐C produced by steroid‐treated MSCs partially reverses the immunosuppression of MSCs on CD8^+^ T cells. A) iNOS deficiency in MSCs partially inhibited the reversal of the immunosuppression of MSCs by Dex. CD8^+^ T cells were isolated and co‐cultured with Ctrl‐MSCs or iNOS^−/−^ MSCs plus anti‐CD3 and anti‐CD28, with or without Dex (10 ng mL^−1^) for 48 h. Cell proliferation was assayed by ^3^H‐thymidine incorporation. B) Venn diagram showing the up‐regulated genes among MSCs of each group. Four groups were presented: MSCs (G1), MSCs with Dex treatment (G2), MSCs co‐cultured with CD8^+^ T cells (G3), and MSCs co‐cultured with CD8^+^ T cells with Dex treatment (G4). C) Heatmap showing the genes with significant change in MSCs of G4, compared to G1 to G3. D,E) mRNA expressions of *Vegfc* (D) and *Vegfd* (E) in MSCs. MSCs were treated with the supernatant of splenocytes activated by anti‐CD3 and anti‐CD28, with or without addition of Dex (10 ng mL^−1^) for 24 h. F) Cell proliferation of CD8^+^ T cells co‐cultured with Ctrl‐MSCs or shVegfc‐MSCs plus anti‐CD3 and anti‐CD28, with or without Dex. Cell proliferation was assayed by ^3^H‐thymidine incorporation. Data are presented as mean ± SEM. n.s: no significance, ^*^ *p* < 0.05 and ^**^ *p* < 0.01.

To parse out the key factor(s) through which Dex‐stimulated MSCs exert the promotion effect on CD8^+^ T cell response, we employed an in vitro co‐culture system to recapitulate the interaction between MSCs and CD8^+^ T cells. Our previous studies have demonstrated that the immune promotion of MSCs could occur when IFN‐*γ* and TNF*α* are at relatively low levels,^[^ ^16^ ^]^ or in the presence of immunosuppressive agents, TGF‐*β*,^[^ ^17^ ^]^ CsA^[^ ^14^ ^]^ or steroids.^[^ ^13^ ^]^ Under this scenario, iNOS was the switch to turn on/off MSCs into either immunosuppression or immune promotion.^[^ ^5^ ^]^ Using MSCs derived from iNOS^−/−^ mice, we found that iNOS deficient MSCs could partially reverse the immunosuppression on CD8^+^ T cells in the presence of Dex (**Figure** **2A**), indicating that there are other factor(s) to promote CD8^+^ T cell response.

Using the in vitro co‐culture system of MSCs and CD8^+^ T cells with or without Dex, we conducted microarray analysis to profile gene expression under these conditions. The gene expression profiles were compared among MSCs (G1), MSCs with Dex treatment (G2), MSCs co‐cultured with CD8^+^ T cells (G3), and MSCs co‐cultured with CD8^+^ T cells in the presence of Dex (G4). As compared to G1, G2, and G3, respectively, there are 1770, 1765, and 200 genes upregulated in G4. We further used the Venn Diagram analysis and identified a core signature of 22 genes shared among these groups (Figure 2B). This set of genes was enriched in biological processes related to vascular endothelial growth and immune cell migration. Among them, VEGF‐C, a paracrine factor, was up‐regulated by 5.23‐folds (Figure 2C; Figure S2A, Supporting Information).

To verify the specificity of elevation in VEGF‐C, we evaluated the mRNA expressions of all members of the VEGF family, including *Vegfa, Vegfb, Vegfc*, and *Vegfd*. We verified that *Vegfc* was specifically upregulated in MSCs in the presence of Dex and T cell supernatant, but only minimally increased when MSCs were treated with either Dex alone or just T cell supernatant (Figure S2B–E, Supporting Information). Notably, when activated T cell supernatant and Dex were added together, there was a synergetic induction of *Vegfc* in MSCs (Figure 2D). In contrast, *Vegfd* was induced by Dex only and no synergistic effect could be detected when T cell supernatant was added (Figure 2E) (the specific factor in the T cell supernatant will be defined below). We therefore questioned whether the upregulation of VEGF‐C in MSCs co‐cultured with T cells and Dex accounts for their promotion on CD8^+^ T cells. To this end, we transfected MSCs with *Vegfc* shRNA or a scrambled control and found that knockdown of *Vegfc* in MSCs significantly reversed their immune promotion effect on activated CD8^+^ T cells in the presence of Dex (Figure 2F; Figure S2F, Supporting Information). Together, these results verified that VEGF‐C is a key factor for mediating the immune promotion effect of MSCs in the presence of steroids.

### 2.3 VEGF‐C Exacerbates aGvHD Progression

**Figure 3:**
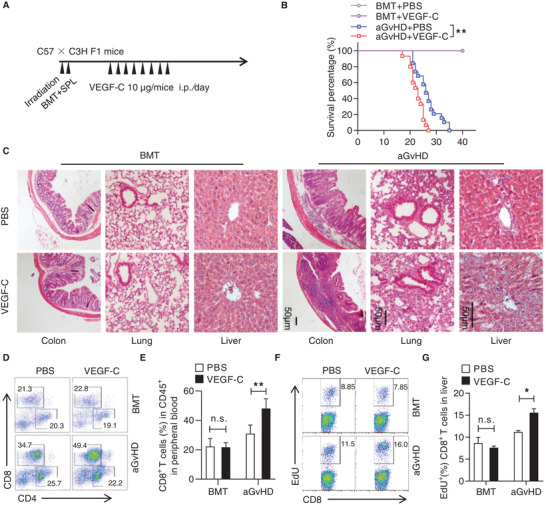
VEGF‐C exacerbates aGvHD progression. A) Experimental scheme of the establishment of aGvHD model for investigating the effect of VEGF‐C. Lethally irradiated C3H × C57BL/6 F1 mice were adoptively transferred with bone marrow cells (5 × 10^6^) and splenocytes (5 × 10^7^) from C57BL/6 mice to induce aGvHD. On day 7 post aGvHD induction, mice were i.p. injected with VEGF‐C at 10 µg per mouse. For the bone marrow transplantation (BMT) group, recipient mice were adoptively transferred with bone marrow cells (5 × 10^6^), with or without VEGF‐C administration. B) Survival curves of aGvHD mice treated with PBS and VEGF‐C (BMT + PBS: *n =* 5, BMT + VEGF‐C: *n =* 5, aGvHD + PBS: *n =* 19, and aGvHD + VEGF‐C: *n =* 15). C) Hematoxilin & eosin staining of the liver, lung, and colon of aGvHD mice with PBS or VEGF‐C treatment. Scale bar: 50 µm. D,E) Flow cytometric analysis of the percentages of CD4^+^ T cells and CD8^+^ T cells in the peripheral blood of aGvHD mice with PBS or VEGF‐C treatment. F,G) Flow cytometric analysis of EdU incorporation in CD8^+^ T cells from the liver of aGvHD mice with PBS or VEGF‐C treatment. Data are presented as mean ± SEM. n.s: no significance, ^*^ *p* < 0.05 and ^**^ *p* < 0.01.

Having identified that VEGF‐C is critical for Dex‐induced immune promotion by MSCs, we then tested its function in aGvHD progression. We found that mice received recombinant VEGF‐C injection exhibited more severe aGvHD, in comparison to the control group (**Figure** **3A**,B). Histological analysis showed more leukocyte infiltrations in the liver, lung, and intestine in VEGF‐C‐treated aGvHD mice, accompanied by severe tissue damage, such as hepatocellular edema, widespread septal thickening in the lung, and reduced crypt cells in the intestine (Figure 3C). However, VEGF‐C administration showed no influence in the BMT group, suggesting that VEGF‐C exerted effects on inflammatory responses (Figure 3B,C). Indeed, by analyzing T cell subsets in peripheral blood, we found that both the percentage and the number of CD8^+^ T cells were increased dramatically in aGvHD mice with VEGF‐C administration (Figure 3D,E; Figure S3C,D, Supporting Information). No significant change was observed in the CD4^+^ T cell population (Figure 3D). In addition, VEGF‐C administration enhanced the total numbers of lymphocytes, especially CD8^+^ T cells, in the liver (Figure S3A,B, Supporting Information), peripheral blood (Figure S3C,D, Supporting Information), and spleen (Figure S3E,F, Supporting Information) of aGvHD mice. By employing EdU incorporation assay in aGvHD mice, we found that T cell proliferation appeared in liver, and that VEGF‐C mostly promoted CD8^+^ T cell proliferation (Figure 3F,G). Thus, VEGF‐C plays a crucial role in modulating CD8^+^ T cell proliferation and aGvHD progression.

### 2.4 VEGF‐C Promotes CD8^+^ T Cell Proliferation via VEGFR3

**Figure 4:**
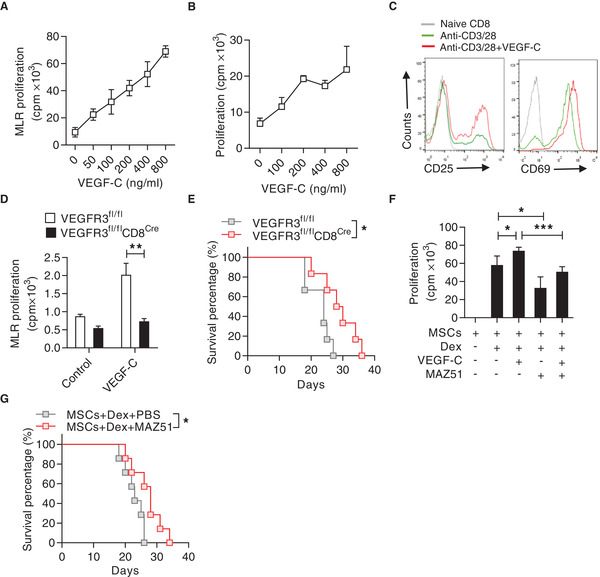
VEGF‐C promotes CD8^+^ T cell proliferation and exacerbates aGvHD. A) Promotion of mixed lymphocyte reaction (MLR) by VEGF‐C. Purified C57BL/6 CD8^+^ T cells (*H2^b^*) were co‐cultured with irradiated BALB/c (*H2^d^*) splenocytes and treated with VEGF‐C at indicated concentrations for 72 h. Cell proliferation was assayed by ^3^H‐thymidine incorporation. B) Proliferation of CD8^+^ T cells promoted by VEGF‐C. CD8^+^ T cells were stimulated by anti‐CD3 and anti‐CD28 in the presence of VEGF‐C at indicated concentrations for 48 h. Cell proliferation was measured by ^3^H‐thymidine incorporation. C) Flow cytometric analysis of the expressions of CD25 and CD69 on CD8^+^ T cells stimulated by anti‐CD3 and anti‐CD28, in presence or absence of VEGF‐C. D) VEGFR3 mediated the promotion effect of VEGF‐C on CD8^+^ T cell proliferation. CD8^+^ T cells were isolated from VEGFR3^fl/fl^ or VEGFR3^fl/fl^CD8^Cre^ mice (C57BL/6) and co‐cultured with irradiated splenocytes from BALB/c mice for 72 h, with or without addition of VEGF‐C (800 ng mL^−1^). Cell proliferation was assayed by ^3^H‐thymidine incorporation. E) Survival curves of aGvHD mice induced by splenocytes from VEGFR3^fl/fl^ and VEGFR3^fl/fl^CD8^Cre^ mice (*n =* 6 for each group). F) Blockade of VEGFR3 signaling by MAZ51 abolished the immune promotion induced by Dex and MSCs. CD8^+^ T cells were isolated and co‐cultured with MSCs plus anti‐CD3 and anti‐CD28, with or without Dex (10 ng mL^−1^), VEGF‐C (800 ng mL^−1^), or MAZ51 (10 µm). Cell proliferation was assessed by ^3^H‐thymidine incorporation. G) Survival rate of aGvHD mice with co‐administration of MSCs and Dex, or the combination of MSCs, Dex, and MAZ51. On day 5, recipients were injected with MSCs (5 × 10^6^) and Dex (2 mg kg^−1^), or MAZ51 at 15 mg per kg per day (*n =* 7). Data are presented as mean ± SEM. ^*^ *p* < 0.05, ^**^p<0.01, and ^***^ *p* < 0.001.

We used a mixed lymphocyte reaction (MLR) system, in which purified C57BL/6 CD8^+^ T cells (*H2^b^*) were mixed with irradiated BALB/c (*H2^d^*) splenocytes, to assess the role of VEGF‐C in CD8^+^ T cell antigenic activation and proliferation. Addition of VEGF‐C to the MLR culture led to a profound enhancement of T cell proliferation in a dose‐dependent manner (**Figure** **4A**). Additionally, VEGF‐C also promoted the proliferation of CD8^+^ T cells induced by anti‐CD3 and anti‐CD28 (Figure 4B). Furthermore, CD8^+^ T cells activated in vitro in the presence of VEGF‐C exhibited higher levels of activation markers CD69 and CD25 than those of the control group (Figure 4C). VEGF‐C had no significant influence on the apoptosis of CD8^+^ T cells (Figure S4A, Supporting Information). In addition, a comparable level of IFN‐*γ* expression was observed between PBS and VEGF‐C treated CD8^+^ T cells upon anti‐CD3 and anti‐CD28 stimulation (Figure S4B, Supporting Information).

We further questioned which VEGF receptor(s) mediates the enhancement of CD8^+^ T cell proliferation induced by VEGF‐C. Previous studies have demonstrated that VEGFR2 and VEGFR3 are the natural receptors for VEGF‐C.^[^ ^18^ ^]^ We thus used flow cytometry to detect the expressions of VEGFR2 and VEGFR3 on CD8^+^ T cells, and we found that VEGFR3 is highly expressed, but not VEGFR2 (Figure S4C, Supporting Information). To further parse out if VEGF‐C promoted CD8^+^ T cell proliferation via VEGFR3, we used the Cre‐Loxp system to specifically delete VEGFR3 in T cells in vivo. VEGFR3 deficient CD8^+^ T cells did not exhibit an enhanced proliferation in response to VEGF‐C as examined in the MLR system (Figure 4D). When aGvHD was induced by transferring bone marrow cells and splenocytes from VEGFR3^fl/fl^CD8^Cre^ mice or VEGFR3^fl/fl^ mice to lethally irradiated C57BL/6 × C3H F1 mice, mice transferred with T cells deficient in VEGFR3 clearly exhibited impaired aGvHD and survived longer than the control mice (Figure 4E). These results demonstrate that VEGF‐C promotes CD8^+^ T cell proliferation and enhances aGvHD through VEGFR3.

As demonstrated above, VEGF‐C was highly produced by MSCs in the presence of Dex and T cell cytokine and VEGF‐C promoted CD8^+^ T cell proliferation by ligating VEGFR3. We sought to determine the role of the VEGF‐C‐VEGFR3 axis in immune promoting effect of MSCs on CD8^+^ T cells, in the presence of Dex. MAZ51, an indolinone with the ability to block tyrosine kinase activity of VEGFR3 signaling, was added to the MSC and CD8^+^ T cell co‐culture system, with or without the addition of Dex or VEGF‐C. We found that blockade of VEGFR3 signaling eliminated the enhancement of CD8^+^ T cell proliferation induced by the co‐existence of MSCs and Dex (Figure 4F). Similarly, MAZ51 administration prolonged the survival time of aGvHD mice which were co‐administrated with MSCs and Dex (Figure 4G). These data demonstrate that steroids abolish the immunosuppression of MSCs on CD8^+^ T cells via VEGF‐C‐VEGFR3 axis.

### 2.5 VEGF‐C Promotes CD8^+^ T Cell Proliferation via PI3K/AKT Activation

**Figure 5:**
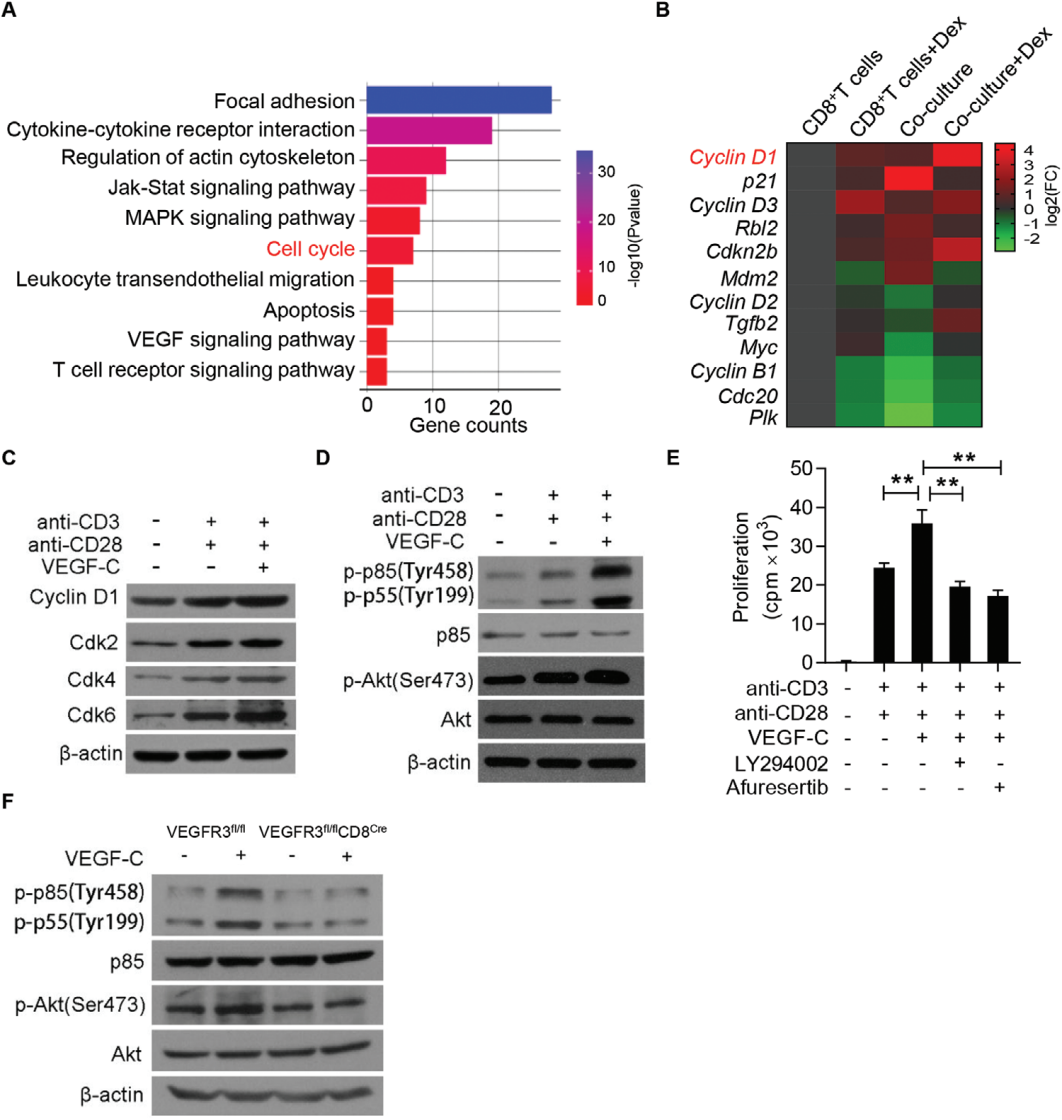
VEGF‐C augments CD8^+^ T cell proliferation via activating the PI3K/AKT signaling pathway. A) GO analysis showing the enriched pathways in CD8^+^ T cells co‐cultured with MSCs plus anti‐CD3 and anti‐CD28, in presence or absence of Dex. B) Heatmap showing genes associated with cell cycle in activated CD8^+^ T cells, with or without the addition of Dex or MSCs. C) Immunoblotting analysis of Cyclin D1, Cdk2, Cdk4, and Cdk6 in lysates of CD8^+^ T cells stimulated with anti‐CD3 and anti‐CD28, with or without addition of VEGF‐C for 24 h. D) Immunoblotting analysis of p85/p55, Akt, and their phosphorylation in lysates of CD8^+^ T cells stimulated with anti‐CD3 and anti‐CD28, with or without the addition of VEGF‐C. E) Inhibition of PI3K/AKT signaling abolished the promotion of VEGF‐C on CD8^+^ T cell proliferation. CD8^+^ T cells were isolated and stimulated with anti‐CD3 and anti‐CD28, with or without VEGF‐C in the presence or absence of LY294002 (20 µm) or Afuresertib (1 µm) for 48 h. Cell proliferation was assessed by ^3^H‐thymidine incorporation. F) Immunoblotting analysis of p85/p55, Akt and their phosphorylation in lysates of CD8^+^ T cells derived from VEGFR3^fl/fl^ and VEGFR3^fl/fl^CD8^Cre^ mice. Data are presented as mean ± SEM. ^**^ *p* < 0.01.

In order to elucidate the molecular mechanisms of the enhanced proliferation of CD8^+^ T cells, RNA‐seq was first performed in CD8^+^ T cells co‐cultured with MSCs, in the presence of Dex or not. Pathways related to focal adhesion, cytokine–cytokine receptor interaction, and regulation of actin cytoskeleton were highly enriched in CD8^+^ T cells co‐cultured with MSCs in the presence of Dex, further pinpointing their strong proliferation potentials (**Figure** **5A**). Detailed analysis on genes related to cell proliferation demonstrated that 12 differentially expressed genes were shown among CD8^+^ T cells and CD8^+^ T cells within the MSCs co‐culture system with or without the addition of Dex. Among these genes, we found that Cyclin D1 was the most upregulated gene in CD8^+^ T cells co‐cultured with MSCs with the addition of Dex (Figure 5B). We further validated that VEGF‐C enhanced the expressions of Cyclin D1 and cyclin‐dependent protein kinases (CDKs), including CDK2, CDK4, and CDK6, in activated CD8^+^ T cells triggered by the engagement of TCR and CD28 (Figure 5C). These results suggest that VEGF‐C promotes the expression of proteins related to G1‐S phase transition and boosts CD8^+^ T cell proliferation.

We next questioned if VEGFR3 signaling impacts proximal TCR signaling. We first assessed the effect of VEGF‐C on CD8^+^ T cell proliferation induced by phorbol‐12‐myristate‐13‐acetate (PMA) and ionomycin, an activation strategy to bypass the requirement of TCR engagement and directly activate protein kinase C and calcium. No significant differences were observed in the expressions of p‐p85/p‐p‐55 and CDKs which are closely related to T cell activation and proliferation, indicating that VEGF‐C may regulate the proximal TCR signaling (Figure S5A, Supporting Information). Among the earliest signaling events in T cells initiated by TCR and CD28 activation, PI3K activity is necessary to catalyze the conversion of phosphatidylinositol (4,5) bisphosphate (PIP2) to phosphatidylinositol (3,4,5) trisphosphate (PtdIns (3,4,5) P3), a secondary signaling molecule that regulates the activity of the serine‐threonine protein kinase B, also known as AKT.^[^ ^19^ ^]^ We found that addition of VEGF‐C enhanced PI3K/AKT activity in CD8^+^ T cells initiated by anti‐CD3 and anti‐CD28, but not PMA and ionomycin (Figure 5D; Figure S5A, Supporting Information). Thus, the enhancement of VEGF‐C on CD8^+^ T cell proliferation is related to PI3K/AKT activity.

We further elucidated if the augmented activity of PI3K/AKT is responsible for the enhancement of CD8^+^ T cell proliferation induced by VEGF‐C. We then used LY294002, a pan‐PI3K inhibitor that suppressed the AKT activity, and Afuresertib, an AKT inhibitor, to treated CD8^+^ T cells and found that blockade of either PI3K or AKT abrogated the promotion effect of VEGF‐C on CD8^+^ T cell proliferation (Figure 5E). As a comparison, we tested the effect of VEGF‐C on CD4^+^ T cells. We first analyzed the expression of VEGFR3 on CD4^+^ T cells and found that it was not detectable (Figure S5B, Supporting Information). Unlike CD8^+^ T cells, the enhancement of Cyclin D1 induced by MSCs and Dex was not observed in CD4^+^ T cells (Figure S5C, Supporting Information). Additionally, VEGF‐C did not increase p‐p85/p‐p55, p‐Akt, and Cyclin D1 expressions in activated CD4^+^ T cells (Figure S5D, Supporting Information). Therefore, VEGF‐C induces immune promotion in CD8^+^ T cells via regulating PI3K/AKT, but not in CD4^+^ T cells.

A prediction from these observations is that activation of VEGFR3 signaling in CD8^+^ T cells can promote the phosphorylation of PI3K/AKT signaling and T cell activation. It is well‐known that VEGFR3 binds and phosphorylates p85*α* to activate PI3K and AKT.^[^ ^20^ ^]^ Using VEGF‐C to treat CD8^+^ T cells isolated from VEGFR3^fl/fl^ and VEGFR3^fl/fl^CD8^Cre^ mice, we found that VEGFR3 deficiency in CD8^+^ T cells completely blocked the phosphorylation of p85 and p55, as well as AKT (Figure 5F). We also found that there were no obvious differences in CD8^+^ T cell activation and apoptosis induction in VEGFR3^fl/fl^ and VEGFR3^fl/fl^CD8^Cre^ mice (Figure S5E,G, Supporting Information). Together, these results indicate that ligation of VEGFR3 with VEGF‐C augments CD8^+^ T cell proliferation in a PI3K/AKT dependent manner.

### 2.6 Steroids Potentiate TNF*α*‐induced VEGF‐C Expression in MSCs

**Figure 6:**
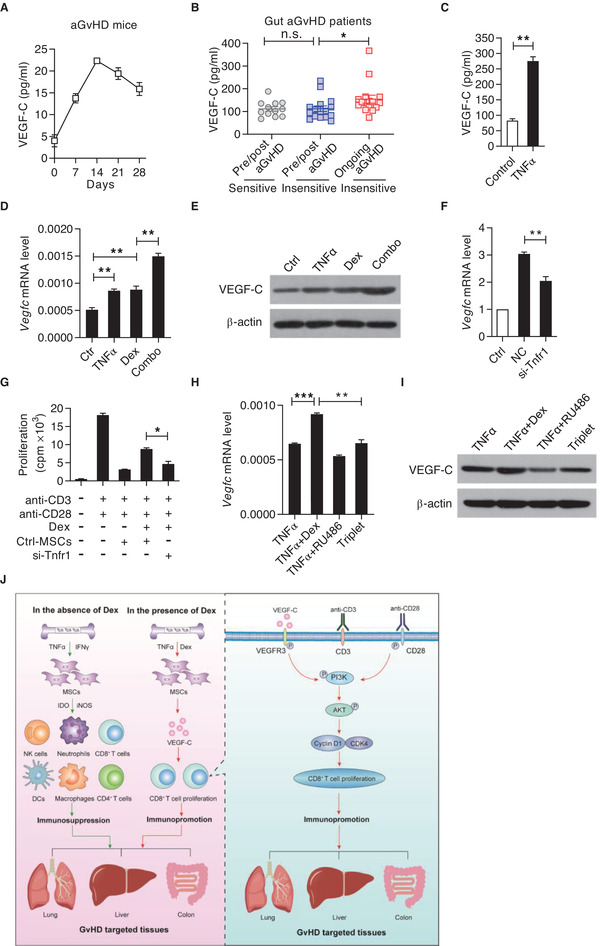
Steroids potentiate TNF*α*‐induced VEGF‐C expression in MSCs. A) VEGF‐C levels in mouse plasma during aGvHD progression at indicated time points as assayed by ELISA. B) VEGF‐C levels in the plasma of aGvHD patients sensitive or resistant to steroid treatment. Pre/post aGvHD (sensitive) (*n =* 12), pre/post aGvHD (insensitive) (*n =* 16), and ongoing aGvHD (insensitive) (*n =* 16). C) VEGF‐C levels in the supernatant of MSCs with or without TNF*α* stimulation as assayed by ELISA. D,E) Expressions of VEGF‐C mRNA (D) and protein (E) in MSCs treated with Dex (10 ng mL^−1^), TNF*α* (10 ng mL^−1^), or their combination for 24 h. F) *Vegfc* mRNA expression in MSCs stimulated with or without TNF*α*. *Tnfr1* expression was knockdown using siRNA. G) MSCs with *Tnfr1* knockdown impaired Dex‐induced CD8^+^ T cell proliferation. Anti‐CD3 and anti‐CD28 activated CD8^+^ T cells were co‐cultured with Ctrl‐MSCs or siRNA‐*Tnfr1* MSCs with or without Dex. Cell proliferation was assessed by ^3^H‐thymidine incorporation. H,I) Expressions of *Vegfc* mRNA (H) and protein (I) in MSCs stimulated with TNF*α* (3 ng mL^−1^), in presence or absence of Dex (50 ng mL^−1^) with or without RU486 (1 µm) for 12 h. J) Graphical abstract. The immunoregulatory property of MSCs is highly plastic. The final read‐out of MSCs depends on the strength and types of inflammatory cytokine and the presence of immunosuppressants. In the presence of IFN‐*γ* and TNF*α*, MSCs elicit immunosuppressive property to alleviate aGvHD progression through suppressing the function of a panel of immune cells, including NK cells, neutrophils, CD8^+^ T cells, CD4^+^ T cells, macrophages, and DCs. However, in the presence of Dex and TNF*α*, MSCs promote immune response via enhancing VEGF‐C‐mediated CD8^+^ T cell response. Mechanistically, Dex and TNF*α* synergistically induce MSCs to produce abundant VEGF‐C. Subsequently, VEGF‐C binds to VEGFR3 on CD8^+^ T cells to augment PI3K/AKT signaling and elevate Cyclin D1 expression, eventually enhancing CD8^+^ T cell response and exacerbating aGvHD progression. Data are presented as mean ± SEM. n.s: no significance, ^*^ *p* < 0.05, ^**^ *p* < 0.01, and ^***^ *p* < 0.001.

To explore the mechanisms of enhanced VEGF‐C expression induced by steroids in vivo, we first detected the dynamic changes of VEGF‐C in mice suffering from aGvHD and in aGvHD patients, sensitive or insensitive to steroid treatment. The concentrations of VEGF‐C in the plasma of aGvHD mice were gradually increased along with the aGvHD progression (**Figure** **6A**). More importantly, aGvHD patients resistant to steroid treatment exhibited much higher levels of VEGF‐C in the plasma than in those patients recovered from aGvHD, either sensitive or insensitive to steroid treatment (Figure 6B). Such dramatic changes indicated that VEGF‐C can be induced by inflammation in vivo and its enhanced expression is associated with the resistance to steroids.

We then determined the key factor(s) responsible for VEGF‐C expression in MSCs. To achieve this, a panel of inflammatory cytokines and TLR agonist was tested for their ability to induce VEGF‐C expression. Consistent with the well‐known role of TLR agonist in VEGF‐C expression in macrophages,^[^ ^20^ ^]^ lipopolysaccharide (LPS) significantly induced the production of VEGF‐C in MSCs (Figure S6A, Supporting Information). Similar effect can be achieved by TNF*α* alone (Figure 6C). However, upregulation of VEGF‐C cannot be detected in IFN‐*γ*‐stimulated MSCs (Figure S6A, Supporting Information). In activated CD8^+^ T cells, we also found that there was a dramatic increase of TNF*α* level in the T cell supernatant (Figure S6B, Supporting Information), suggesting that TNF*α* produced by CD8^+^ T cells could induce VEGF‐C expression in MSCs.

Based on these results, we next asked whether Dex augments inflammation‐induced VEGF‐C expression in MSCs. We measured the mRNA level of *Vegfc* in MSCs treated with IFN‐*γ*, TNF*α*, or LPS, in addition to different dosages of Dex (Figure S6C, Supporting Information). We found that *Vegfc* mRNA expression can be induced by Dex to a comparable level of TNF*α*‐stimulated MSCs (Figure 6D; Figure S6D, Supporting Information). Additionally, only *Vegfc* expression was induced by the combination of TNF*α* and Dex, but not *Vegfd* expression (Figure 6D,E; Figure S6E, Supporting Information). Having confirmed that MSCs mainly express *Tnfr1* (Figure S6F, Supporting Information), we knocked down *Tnfr1* in MSCs with small interfering RNA (siRNA). Knockdown of *Tnfr1* decreased the mRNA level of *Vegfc* in MSCs upon TNF*α* treatment (Figure 6F; Figure S6G, Supporting Information). Furthermore, *Tnfr1* knockdown partially diminished the immune promotion of MSCs on CD8^+^ T cells in the presence of Dex (Figure 6G). To verify if Dex elicited response is through interacting with glucocorticoid receptor (GR),^[^ ^21^ ^]^ RU486, an antagonist of intracellular receptor GR, was applied to test the ability of Dex in promoting VEGF‐C expression in MSCs. We found that RU486 completely reversed the augmentation of VEGF‐C expression in Dex‐treated MSCs (Figure 6H,I). These data demonstrate that Dex induces MSCs to express VEGF‐C and can act synergistically with TNF*α*.

## 3 Discussion

Most of what we understood about the immunosuppression of MSCs stemmed from the exploration of their function in inflammatory disease treatments. Inflammatory cytokines are indispensable for MSCs to gain the immunosuppressive capacity. However, inflammatory cytokines enable MSC immunosuppression only at high amounts. When inflammatory cytokines are sufficiently low, MSCs can in fact promote immune response. Although this plasticity of MSC‐mediated immunoregulation provides an explanation for the conditions that MSC administration cannot improve the diseases in certain conditions, the molecular mechanisms remain elusive. Here, we demonstrated that MSC‐based aGvHD treatment can be abolished by steroid co‐administration, which was found to synergistically augment the production of VEGF‐C by MSCs in the presence of TNF*α*. VEGF‐C binding to VEGFR3 specifically enhanced CD8^+^ T cell response, thus profoundly exacerbating aGvHD progression.

These findings provide experimental explanation for the controversial clinical outcomes of MSC application in GvHD patients when steroids are co‐administered,^[^ ^1^, ^3^ ^]^ highlighting that the undesirable influence of the interaction between MSCs and steroids in aGvHD treatment. The current interest in rushing in to apply MSCs to inflammatory disease was all started by a successful application of MSCs in the treatment of refractory GvHD.^[^ ^3^ ^]^ However, in a phase 3 clinical trial, MSC administration to patients with aGvHD fails to reach its primary endpoint.^[^ ^15^ ^]^ There are several possibilities to explain the variations in the outcomes. Many factors could affect the efficacy of MSCs in aGvHD treatment, such as MSC manufacture procedures, timing of MSC administration, types of concurrent therapy and the heterogeneity of aGvHD patients. Of note, inflammation should be thoroughly considered when MSCs are applied. In steroid and CsA refractory GvHD patients, strong inflammation and the associated high levels of inflammatory cytokines could license MSCs to gain the immunosuppressive capacity, thereby extinguishing the inflammatory responses. In the context of aGvHD, steroids are the first‐line choice to downregulate inflammatory responses. Such actions may decrease the inflammation levels to the status that cannot fully initiate the immunosuppression of MSCs. Thus, the plasticity of immunoregulation by MSCs in the context of inflammation conditions should be critically considered when applied to treat inflammatory diseases.

It is well‐known that iNOS (in mouse) and IDO (in human) are the “on–off” switch of the plasticity of the immunoregulation by MSCs.^[^ ^22^ ^]^ Upregulation of iNOS or IDO in MSCs by inflammatory cytokines in sufficient amounts can strengthen the immunosuppression of MSCs.^[^ ^22^ ^]^ Blockade of iNOS or IDO diminishes the immunosuppression of MSCs, and in some cases, enhances T cell proliferation.^[^ ^11^, ^22^ ^]^ Indeed, we observed a partially reversed immune promotion of iNOS^−/−^ MSCs on CD8^+^ T cells, in presence of Dex, indicating that other molecule(s) is involved in mediating the immune promotion by MSCs stimulated with Dex. We found that VEGF‐C produced by MSCs treated with Dex exerted strong immune promoting function and exacerbated aGvHD progression. Interestingly, VEGF‐C alone could enhance aGvHD progression. Thus, these results provide a novel mechanism of steroid interference on the immunosuppression of MSCs and offer insights for the application of MSCs in aGvHD patients. Of note, when we analyzed the VEGF‐C levels in aGvHD patients with or without responses to steroid treatment, we found that the enhanced VEGF‐C production is associated with feeble efficacy in treating aGvHD by steroids.

Although various studies have demonstrated that MSC administration improved aGvHD in both preclinical and clinical studies by directly or indirectly regulating T cells, the mechanisms related to MSCs and the specific T cell subpopulation remain elusive. By detailed analysis of T cell populations, CD8^+^ T cells, but not CD4^+^ T cells, were found to be the major cell population in mediating the worsening of aGvHD by concurrent administration of MSCs and steroids. Thus, the understanding of the relationship between MSCs and CD8^+^ T cells is crucial in dissecting the complicated interaction between MSCs and inflammation during the pathogenesis of various inflammatory diseases.

Most importantly, we found that VEGF‐C produced by MSCs in the presence of TNF*α* and steroids aggravated aGvHD via enhancing the activation of CD8^+^ T cells. Our data from mice with VEGFR3 specific deletion in T cells suggested that activation of VEGFR3 dramatically promoted CD8^+^ T cell activation through enhancing the PI3K/AKT signaling. This finding contrasts with the results from a study in macrophages from Chy mice, which reported that activation of VEGFR3 by VEGF‐C in macrophages suppressed inflammation.^[^ ^20^ ^]^ It is important to point out that, in our study, we compared the effect of the axis of VEGF‐C/VEGFR3 on CD4^+^ and CD8^+^ T cells, both are major populations of adaptive immune response. Distinct with the augmentation of VEGF‐C on CD8^+^ T cell activation and proliferation via VEGFR3, the effect on CD4^+^ T cells was not observed. The lack of the expression of VEGFR3 on CD4^+^ T cells may explain the discrepancy between CD4^+^ and CD8^+^ T cells. Further studies on the differences of VEGFR3 signaling in modulating T cells and macrophages, as well as other subtype of immune cells may help to decipher the function of VEGF‐C/VEGFR3 in the context of distinct inflammatory diseases.

Collectively, our findings show that VEGF‐C produced by TNF*α* and steroid stimulated‐MSCs enhances CD8^+^ T cell response. Such effect occurs when aGvHD is treated concurrently with MSCs and steroids and aGvHD patients that are resistant to steroid treatment (Figure 6J). We believe that our findings not only demonstrate the role of VEGF‐C/VEGFR3 in regulating CD8^+^ T cell proliferation, but also reveal a key principle of MSC application in treatment of aGvHD.

## 4 Experimental Section

##### Study Design

This study was initiated to explore the interaction between MSCs and steroids in aGvHD. To achieve this aim, both experimental aGvHD mouse model and human plasma samples were used. First, a mouse aGvHD model and a T cell co‐culture system to investigate how steroids interfere the immunosuppression by MSCs were employed. Using RNA sequencing analysis, it was identified that VEGF‐C was responsible for the Dex‐induced immune promotion by MSCs. Next, the influence of VEGF‐C on CD8^+^ T cell proliferation and aGvHD progression was examined. In order to evaluate the signaling pathway involved in VEGF‐C‐treated CD8^+^ T cells, RNA sequencing and western blotting analysis were used. Finally, to better understand the clinical relevance between the VEGF‐C level and human GvHD progression, the levels of VEGF‐C in the plasma of glucocorticoid sensitive or insensitive patients were detected. The use of human blood samples was approved by the Ethic Committee of the First Affiliated Hospital of Soochow University. All samples were collected under the informed consent.

##### Mice

C57BL/6, BALB/c, C3H/HeJCr mice were purchased from the Shanghai Laboratory Animal Center of Chinese Academy of Science (Shanghai, China). CD8‐Cre mice were purchased from the Jackson Laboratory (Bar Harbor, ME). VEGFR3^fl/fl^ mice on the C57BL/6 background were generated from Biomodel (Shanghai Research Center for Model Organisms). Briefly, the exon 2 of VEGFR3 was modified by the insertion of two loxP sites that enable to excise the floxed gene segment through Cre‐mediated recombination. Homologous recombination was employed to generate VEGFR3^fl/fl^ mice. First, the in‐fusion cloning method was used to construct donor vector. Then, Cas9 mRNA, gRNA, and donor vector were microinjected into the fertilized egg of C57BL/6J mice to obtain F0 mice. The F0 mice were mated with C57BL/6 mice to obtain VEGFR3^fl/fl^ mice. To obtain mice with VEGFR3 specific deletion in CD8^+^ T cells, VEGFR3^fl/fl^ mice were bred with CD8‐Cre mice, with inducible Cre recombinase expression in CD8^+^ T cells. Mice were maintained under specific pathogen‐free condition. Animals were matched for age and gender in each experiment. All animal procedures were approved by the Institutional Animal Care and Use Committee of the Institute of Nutrition and Health, Shanghai Institutes for Biological Sciences of Chinese Academy of Sciences.

##### Reagents

Recombinant mouse IFN‐*γ* and TNF*α* were purchased from R&D Systems (Minneapolis, MN, USA). Dex sodium phosphate was purchased from Suzhou No. 6 Pharmaceutical Factory (Suzhou, China). RU486 and LPS were from Sigma Aldrich (St. Louis, MO, USA). Purified antibodies specific for CD3 and CD28, and fluorescent labeled antibodies specific for CD45, CD3, CD4, CD8, CD25, CD69, and IFN‐*γ* were from eBioscience (San Diego, CA, USA). MAZ51 was purchased from Millipore (Billerica, MA, USA). LY294002 and Afuresertib were purchased from Selleck (Houston, TX, USA). EdU was purchased from Invitrogen (Carlsbad, CA, USA). Mouse *Vegfc* shRNA lentiviral particles and control particles were prepared by GenePharma (Shanghai, China) with the following sequences:

shRNA 1^#^ (LV3‐Vegfc‐mus‐637): 5′‐GCCACGTGAGGTGTGTATAGA‐3′,

shRNA 2^#^ (LV3‐Vegfc‐mus‐1400): 5′‐GCGAATCGACTGAAGCATTGT‐3′.

##### Cells

MSCs were generated from mouse bone marrow. Bone marrow cells were harvested and cultured in Dulbecco's modified Eagle's medium supplemented with 10% fetal bovine serum, 2 mm glutamine, 100 U mL^−1^ penicillin, and 100 µg mL^−1^ streptomycin (all from Invitrogen, Carlsbad, CA, USA). After 8 h, non‐adherent cells were removed and adherent cells were maintained with medium replenishment every 2–3 days. Using a panel of surface markers, MSCs were determined. Positivity in markers of hematopoietic stem cells and lineage cells was used to identify non‐MSCs. The stemness was defined by the capacity to differentiate into adipocytes, osteoblasts, and chondrocytes.

Splenocytes were harvested from mice mentioned above. CD4^+^ or CD8^+^ T cells were isolated using magnetic cell sorting kit (Miltenyi, Bergisch Gladbach, Germany). T cells were maintained in RPMI‐1640 medium supplemented with 10% fetal bovine serum, 2 mm glutamine, 100 U mL^−1^ penicillin, 100 µg mL^−1^ streptomycin, 1 mm sodium pyruvate, and 55 µm 2‐mercaptoethanol.

##### Patient Plasma Samples

The plasma samples of BMT leukemia patients diagnosed with aGvHD were collected and used for analysis (YouQin Medical Laboratory). Based on their response to glucocorticoids, patients were divided into sensitive and insensitive groups. Patients showed progression for 3 days, or no improvement lasting for at least 7 days after treatment with 2 mg kg^−1^ per day prednisolone were classified into the insensitive group. The plasma of glucocorticoid sensitive or insensitive patients were used for VEGF‐C protein level detection.

##### Mixed Lymphocyte Reaction

In the MLR system, responder T cells were co‐cultured with allogeneic lymphocytes as a stimulator. Splenocytes were harvested from C57BL/6 mice and their CD8^+^ T cells were purified as responder cells. Splenocytes from BALB/c mice were irradiated at a dose of 14 Gy to prevent cell proliferation, and were used as a stimulator to generate one‐way MLR. The MLR was performed by seeding responder cells (2 × 10^5^ per well) in 96‐well plates and co‐cultured with irradiated allogeneic cells (8 × 10^5^ per well) from BALB/c mice. Recombinant VEGF‐C (Sino biological, Beijing, China) was added to each well with the final concentration at 100–800 ng mL^−1^.

##### ^3^H‐Thymidine Incorporation Assay

T cell proliferation was assessed by the addition of 0.5 µCi of ^3^H‐methyl‐thymidine (Perkin Elmer, Boston, MA, USA) during the final 6 h of culture followed by scintillation counting of incorporated ^3^H‐thymidine using a Wallac Microbeta scintillation counter (Perkin‐Elmer, Waltham, MA, USA).

##### Mouse aGvHD Model

Eight to ten weeks old C57BL/6 × C3H F1 mice were lethally irradiated (13 Gy). After resting for 24 h, these mice were i.v. injected with nucleated bone marrow cells (5 × 10^6^) and splenocytes (5 × 10^7^) isolated from C57BL/6 mice. On days 5 and 7 following BMT, the recipients were administrated with MSCs (5 × 10^6^) derived from C57BL/6 mice via the tail vein. Some groups were also injected with Dex (2 mg kg^−1^) 3 times per week. Mice were observed and euthanized upon becoming moribund, thus recording survival time.

##### Flow Cytometric Analysis

Cell surface markers and intracellular cytokines were stained according to the eBioscience flow cytometric protocols. Briefly, cells were collected and washed with PBS. For surface markers staining of CD45, CD3, CD4, CD8, CD25, CD69, CD11b, CD11c, CD34, Sca‐1, CD44, CD140a, CD25, CD69 (eBioscience), VEGFR2, and VEGFR3 (R&D), cells were resuspended in PBS containing 0.5% BSA and incubated with fluorochrome‐conjugated antibodies at room temperature for 30 min. For intracellular staining, cells were fixed, permeabilized, and then stained with IFN‐*γ* (eBioscience). Cell samples were analyzed using a BD FACS Calibur flow cytometer (BD Biosciences). FlowJo software was used for data analysis.

##### EdU Incorporation Assay

To assess cell proliferation, the EdU incorporation assay was carried out with the Click‐iT EdU assay kit (Life Technologies, USA). Mice were i.p. injected with 10 µg EdU per gram of body weight and were euthanized 3 h later. The liver was harvested and homogenized by pressing through cell strainer (70 µm, BD, Franklin Lakes, NJ, USA). After washing, cells were resuspended in 35% Percoll (GE‐healthcare, Uppsala, Sweden) solution and then layered on 70% Percoll followed by centrifugation at 2000 rpm for 20 min at room temperature. Lymphocytes were collected from the interface and washed twice with RPMI 1640 medium. The Click‐iT reaction was performed and analyzed flow cytometrically according to the manufacturer's instruction. Cells were evaluated on a FACS Calibur and the data were analyzed by FlowJo software.

##### Quantitative PCR and Microarray Analysis

Total RNA was extracted using the Trizol kit (Invitrogen). First‐strand cDNA synthesis was performed using a cDNA Synthesizing Kit (Takara). Genes of interest were quantified by Real‐Time PCR (7900HT by Applied Biosystems, Foster City, CA, USA), using SYBR Green Master Mix (Roche Diagnostics, Indianapolis, IN, USA) as an indicator. Total amount of mRNA was normalized to endogenous *β*‐actin mRNA. Sequences of PCR primer pairs were as follows: Mouse *β*‐actin, forward 5′‐GCTCTGGCTCCTAGCACCAT‐3′, reversed 5′‐CCACCGATCCACACAGAGTAC‐3′; Mouse *Vegfa*, forward 5′‐GCACATAGAGAGAATGAGCTTCC‐3′, reversed 5′‐CTCCGCTCTGAACAAGGCT‐3′; Mouse *Vegfb*, forward 5′‐GCCAGACAGGGTTGCCATAC‐3′, reversed 5′‐GGAGTGGGATGGATGATGTCAG‐3′; Mouse *Vegfc*, forward 5′‐GAGGTCAAGGCTTTTGAAGGC‐3′, reversed 5′‐CTGTCCTGGTATTGAGGGTGG‐3′; Mouse *Vegfd*, forward 5′‐TTGAGCGATCATCCCGGTC‐3′, reversed 5′‐GCGTGAGTCCATACTGGCAAG‐3′; Mouse *Tnfr1*, forward 5′‐CGATAAAGCCAC ACCCACAA‐3′, reversed 5′‐ACCTTTGCCCACTTTTCACC‐3′; Mouse *Tnfr2*, forward 5′‐GGGCACCTTTACGGCTTCC‐3′, reversed 5′‐GGTTCTCCTTACAGCCACACA‐3′. Microarray was performed by Affymetrix mouse Gene 2.0 ST Array (Affymetrix, Santa Clara, CA, USA).

##### Western Blotting Analysis

Isolated CD8^+^ T cells were lysed using the RIPA lysis buffer (BeyoTime) on ice. Total protein concentration was determined by a BCA Protein assay kit (Bio‐Rad). Protein samples were separated by SDS‐PAGE and transferred onto PVDF membranes, which was then blocked with 5% fat‐free milk for 1 h. Blots were incubated with primary antibodies specific to p‐p85/p‐p55, p85, p‐Akt, Akt, Cyclin D1, Cdk2, Cdk4, and Cdk6 (Cell Signaling Technology, USA) followed by secondary antibodies conjugated with horseradish peroxidase, and the staining was detected with the ECL system (Millipore).

##### Hematoxylin & Eosin Histological Staining

On day 21 following aGvHD induction, the liver, lung, and intestine were collected and prepared for hematoxylin & eosin staining. Tissues were fixed in 4% paraformaldehyde overnight. These tissues were then dehydrated through sequentially treatment with 75% ethanol, 95% ethanol, and 100% ethanol. The tissues were then treated with xylene for 10 min and embedded in paraffin. The samples were then sectioned at 5 µm and stained with hematoxylin and eosin. Micrographs were captured using a Zeiss Observer Z1 (Carl Zeiss, Jena, Germany).

##### Elisa Assay

The levels of VEGF‐C in the supernatant of MSCs, the plasma of aGvHD mice and the plasma of aGvHD patients were determined by ELISA kit purchased from Westang, Shanghai, China. The level of TNF*α* in CD8^+^ T cell supernatant was determined by an ELISA kit purchased from eBioscience.

##### Gene Knockdown Using siRNA and shRNA

*Vegfc* expression was knocked down in MSCs using Vegfc‐targeting shRNA. Mouse *Vegfc* shRNA lentiviral particles and control particles were prepared by GenePharma (Shanghai, China). siRNA for specific knock down *Tnfr1* was purchased from Sangon, Shanghai, China. Sequences of siRNA and shRNA were as follows: shRNA 1# (LV3‐*Vegfc*‐mus‐637): 5′‐GCCACGTGAGGTGTGTATAGA‐3′; shRNA 2# (LV3‐*Vegfc*‐mus‐1400): 5′‐GCGAATCGACTGAAGCATTGT‐3′. *Tnfrsf1a*‐mus‐637 (siRNA1). Sense: 5′‐GGAGAUCUCUCCUUGCCAATT‐3′, Antisense: 5′‐UUGGCAAGGAGAGAUCUCCTT‐3′; *Tnfrsf1a*‐mus‐894 (siRNA2). Sense: 5′‐CCGCUUGCAAAUGUCACAATT‐3′, Antisense: 5′‐UUGUGACAUUUGCAAGCGGTT‐3′. *Tnfrsf1a*‐mus‐1612 (siRNA3). Sense: 5′‐CCUCGAGGCUCUGAGAAAUTT‐3′, Antisense: 5′‐AUUUCUCAGAGCCUCGAGGTT‐3′.

##### Lentivirus Transfection

The lentivirus particles for knockdown of VEGF‐C were purchased from GenePharma. MSCs were infected with the described lentiviral vectors supplemented with 10 mg mL^−1^ polybrene (GeneChem). After transfection, MSCs were selected in medium containing 2 mg mL^−1^ puromycin.

##### Apoptosis Analysis

Annexin V/Propidium iodide staining (Apoptosis Detection Kit, eBioscience) was performed to assess apoptotic and necrotic cells. Briefly, naïve CD8^+^ T cells were isolated from VEGFR3^fl/fl^ and VEGFR3^fl/fl^CD8^Cre^ mice. CD8^+^ T cells were stimulated with anti‐CD3 and anti‐CD28 for 48 h. Staining was carried out according to the manufacturer's instructions. Cells were collected and washed with PBS, then analyzed using a BD FACS Caliber flow cytometer (BD Biosciences). The data were analyzed using the FlowJo software.

##### Statistics

Data are shown as means ± SEM as specified in the figure legends and analyzed with GraphPad Prism 8. The number of mice used per treatment group is indicated as “n” in the corresponding figure legends. Student's *t*‐test (two tailed) and Log‐rank (Mantel–Cox) test were used for statistical analysis. Significant differences are indicated as follows: n.s: no significance, ^*^ *p* < 0.05, ^**^ *p* < 0.01, and ^***^ *p* < 0.001.

## Conflict of Interest

The authors declare no conflict of interest.

## Author contributions

Y.G. and T.Z. contributed equally to this work. Y.G. and T.Z. designed and performed the experiments. X.C., W.C., L.L., L.D., and Y.W. helped conduct experiments. X.H., F.Z., and H.S. assisted in clinical samples. Y.H. provided suggestions for mouse experiments. J.G. and L.S. provided help for revision. Y.W. and Y.S. led the project and wrote the manuscript.

## Supporting information

Supporting Information

## Data Availability

Research data are not shared.

## References

[1] Y. Wang , X. Chen , W. Cao , Y. Shi , Nat. Immunol. 2014, 15, 1009. 25329189 10.1038/ni.3002

[2] Y. Shi , Y. Wang , Q. Li , K. Liu , J. Hou , C. Shao , Y. Wang , Nat. Rev. Nephrol. 2018, 14, 493. 29895977 10.1038/s41581-018-0023-5

[3] K. L.e Blanc , I. Rasmusson , B. Sundberg , C. Gotherstrom , M. Hassan , M. Uzunel , O. Ringden , Lancet 2004, 363, 1439. 15121408 10.1016/S0140-6736(04)16104-7

[4] R. Meisel , A. Zibert , M. Laryea , U. Gobel , W. Daubener , D. Dilloo , Blood 2004, 103, 4619. 15001472 10.1182/blood-2003-11-3909

[5] K. Sato , K. Ozaki , I. Oh , A. Meguro , K. Hatanaka , T. Nagai , K. Muroi , K. Ozawa , Blood 2007, 109, 228. 16985180 10.1182/blood-2006-02-002246

[6] M. E. Groh , B. Maitra , E. Szekely , O. N. Koc , Exp. Hematol. 2005, 33, 928. 16038786 10.1016/j.exphem.2005.05.002

[7] L. Du , L. Lin , Q. Li , K. Liu , Y. Huang , X. Wang , K. Cao , X. Chen , W. Cao , F. Li , C. Shao , Y. Wang , Y. Shi , Cell Metab. 2019, 29, 1363. 30745181 10.1016/j.cmet.2019.01.006

[8] L. Bai , D. P. Lennon , A. I. Caplan , A. DeChant , J. Hecker , J. Kranso , A. Zaremba , R. H. Miller , Nat. Neurosci. 2012, 15, 862. 22610068 10.1038/nn.3109 PMC3427471

[9] E. Sala , M. Genua , L. Petti , A. Anselmo , V. Arena , J. Cibella , L. Zanotti , S. D'Alessio , F. Scaldaferri , G. Luca , I. Arato , R. Calafiore , A. Sgambato , S. Rutella , M. Locati , S. Danese , S. Vetrano , Gastroenterology 2015, 149, 163. 25790743 10.1053/j.gastro.2015.03.013

[10] S. Aggarwal , M. F. Pittenger , Blood 2005, 105, 1815. 15494428 10.1182/blood-2004-04-1559

[11] G. Ren , L. Zhang , X. Zhao , G. Xu , Y. Zhang , A. I. Roberts , R. C. Zhao , Y. Shi , Cell Stem Cell 2008, 2, 141. 18371435 10.1016/j.stem.2007.11.014

[12] M. Sudres , F. Norol , A. Trenado , S. Gregoire , F. Charlotte , B. Levacher , J. J. Lataillade , P. Bourin , X. Holy , J. P. Vernant , D. Klatzmann , J. L. Cohen , J. Immunol. 2006, 176, 7761. 16751424 10.4049/jimmunol.176.12.7761

[13] X. Chen , Y. Gan , W. Li , J. Su , Y. Zhang , Y. Huang , A. I. Roberts , Y. Han , J. Li , Y. Wang , Y. Shi , Cell Death Dis. 2014, 5, e1009. 24457953 10.1038/cddis.2013.537 PMC4040685

[14] S. Inoue , F. C. Popp , G. E. Koehl , P. Piso , H. J. Schlitt , E. K. Geissler , M. H. Dahlke , Transplantation 2006, 81, 1589. 16770249 10.1097/01.tp.0000209919.90630.7b

[15] P. Kebriaei , J. Hayes , A. Daly , J. Uberti , D. I. Marks , R. Soiffer , E. K. Waller , E. Burke , D. Skerrett , E. Shpall , P. J. Martin , Biol. Blood Marrow Transplant. 2020, 26, 835. 31505228 10.1016/j.bbmt.2019.08.029 PMC7060124

[16] W. Li , G. Ren , Y. Huang , J. Su , Y. Han , J. Li , X. Chen , K. Cao , Q. Chen , P. Shou , L. Zhang , Z. R. Yuan , A. I. Roberts , S. Shi , A. D. Le , Y. Shi , Cell Death Differ. 2012, 19, 1505. 22421969 10.1038/cdd.2012.26 PMC3422473

[17] C. Xu , P. Yu , X. Han , L. Du , J. Gan , Y. Wang , Y. Shi , J. Immunol. 2014, 192, 103. 24293629 10.4049/jimmunol.1302164

[18] a) V. Joukov , K. Pajusola , A. Kaipainen , D. Chilov , I. Lahtinen , E. Kukk , O. Saksela , N. Kalkkinen , K. Alitalo , EMBO J. 1996, 15, 1751; 8612600 PMC450088

[19] a) F. Garcon , D. T. Patton , J. L. Emery , E. Hirsch , R. Rottapel , T. Sasaki , K. Okkenhaug , Blood 2008, 111, 1464; 18006698 10.1182/blood-2007-08-108050

[20] Y. Zhang , Y. Lu , L. Ma , X. Cao , J. Xiao , J. Chen , S. Jiao , Y. Gao , C. Liu , Z. Duan , D. Li , Y. He , B. Wei , H. Wang , Immunity 2014, 40, 501. 24656836 10.1016/j.immuni.2014.01.013

[21] N. C. Nicolaides , A. N. Pavlaki , M. A. Maria Alexandra , G. P. Chrousos , in Endotext, (Eds: K. R. Feingold , B. Anawalt , A. Boyce , G. Chrousos , K. Dungan , A. Grossman , J. M. Hershman , G. Kaltsas , C. Koch , P. Kopp , M. Korbonits , R. McLachlan , J. E. Morley , M. New , L. Perreault , J. Purnell , R. Rebar , F. Singer , D. L. Trence , A. Vinik , D. P. Wilson ), MDText.com, Inc., South Dartmouth, MA 2000.

[22] W. Ling , J. Zhang , Z. Yuan , G. Ren , L. Zhang , X. Chen , A. B. Rabson , A. I. Roberts , Y. Wang , Y. Shi , Cancer Res. 2014, 74, 1576. 24452999 10.1158/0008-5472.CAN-13-1656 PMC3959857

