# Supplementary material for: Steroids Enable Mesenchymal Stromal Cells to Promote CD8+ T Cell Proliferation Via VEGF‐C
Source: Adv Sci (Weinh). 2021 May 2;8(12):2003712. doi: 10.1002/advs.202003712 (PMC8224440; doi:10.1002/advs.202003712)
Supplement: Supplementary file 1 — Supporting Information [file ADVS-8-2003712-s001.pdf]

© 2021 Wiley-VCH GmbH

ADVANCED SCIENCE
Open Access

Supporting Information

for *Adv. Sci.,* DOI: 10.1002/advs.202003712

Steroids Enable Mesenchymal Stromal Cells to Promote CD8+ T Cell Proliferation via VEGF-C

Yurun Gan, Tao Zhang, Xiaodong Chen, Wei Cao, Liangyu Lin, Liming Du, Yu Wang, Fei Zhou, Xuefeng He, Yulong He, Jianhe Gan, Huiming Sheng, Lydia Sorokin, Yufang Shi, and Ying Wang**

Supporting Information

Steroids Enable Mesenchymal Stromal Cells to Promote $CD8^+$ T cell Proliferation via VEGF-C

Yurun Gan, Tao Zhang, Xiaodong Chen, Wei Cao, Liangyu Lin, Liming Du, Yu Wang, Fei Zhou, Xuefeng He, Yulong He, Jianhe Gan, Huiming Sheng, Lydia Sorokin, Yufang Shi, and Ying Wang**

Supplementary Figures:

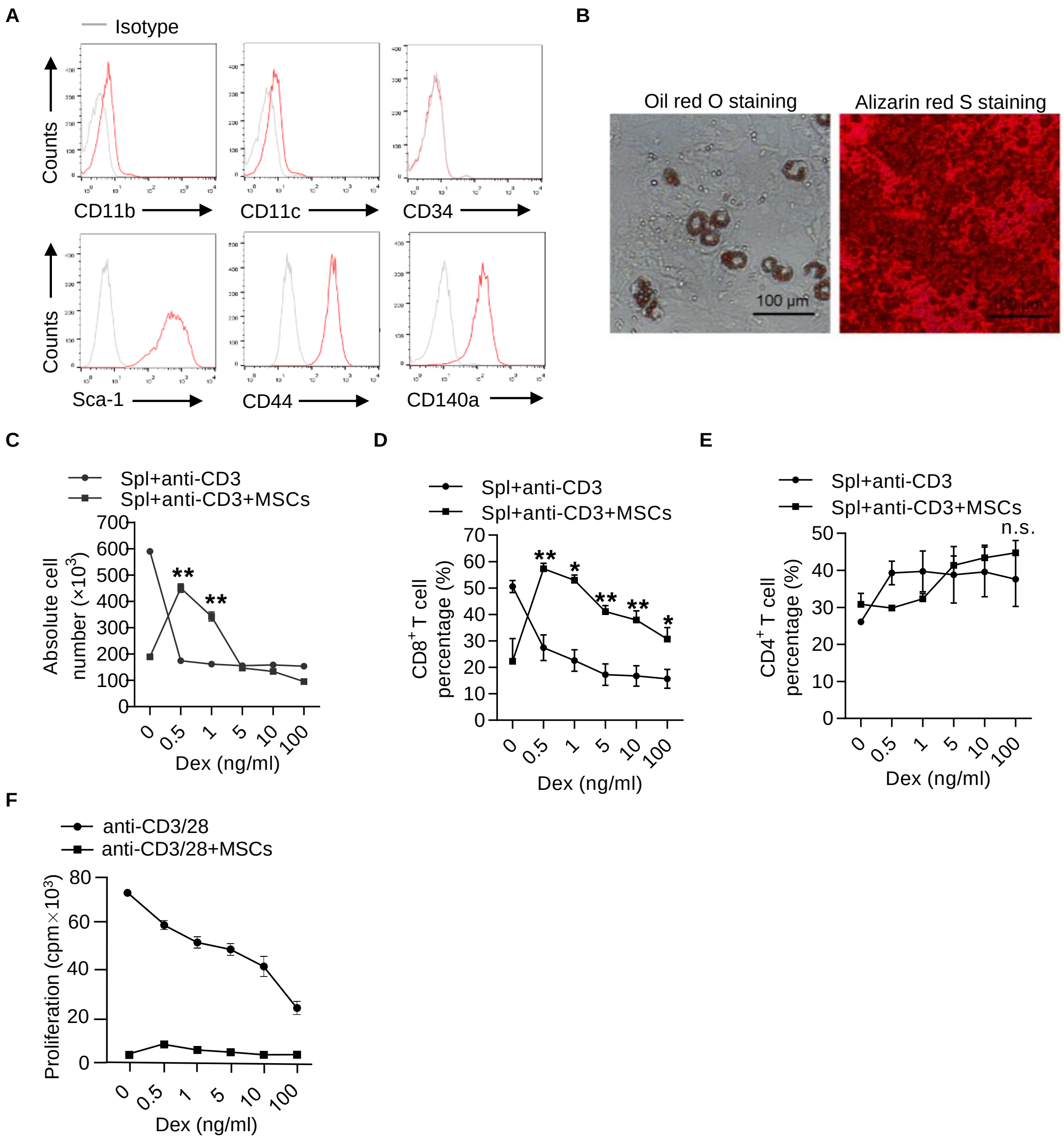

Figure S1. $CD4^+$ T cells are not involved in immune promotion by Dex-treated MSCs. A) Flow cytometric analysis of the expressions of CD11b, CD11c, CD34, Sca-1, CD44 and CD140a in MSCs. B) Oil red O staining and Alizarin red S staining of MSCs cultured in adipogenic and osteogenic differentiation medium for 7 and 21 days, respectively. C) Splenocytes were activated by anti-CD3 and co-cultured with or without MSCs in the presence or absence of Dex at indicated concentrations, and the total cell numbers were counted. D-E) Flow cytometric analysis of the percentages of $CD4^+$ T cells and $CD8^+$ T cells among splenocytes. F) The proliferation of $CD4^+$ T cells. $CD4^+$ T cells isolated from the mouse spleen were stimulated with anti-CD3 and anti-CD28 with or without MSCs, in the presence or absence of Dex at indicated concentrations. Cell proliferation was assessed by ^{3}H-

thymidine incorporation. Data are presented as mean ± SEM. n.s., no significance, $*p < 0.05$ and $**p < 0.01$.

A

Name	Fold change	Description	Cellular Component	gene Symbol
Dtl	5.44	denticleless homolog (Drosophila)	nucleus;chromosome;cytoplasm;centrosome	1456652_at
Vegfc	5.23	vascular endothelial growth factor C	extracellular region ; extracellular space	1419417_at
Gda	4.53	guanine deaminase	intracellular	1435748_at
D17H6S56E-5	3.59	DNA segment, Chr 17, human D6S56E 5	viral capsid; viral envelope	1417822_at
Ccl9	3.59	chemokine (C-C motif) ligand 9	extracellular region;extracellular space	1417936_at
Hist1h2ab	2.86	histone cluster 1, H2ab	nucleosome; nucleus; chromosome	1438009_at
C79407	2.08	expressed sequence C79407	nucleus;nucleolus; cytoplasm	1458374_at
Kif20b	2.01	kinesin family member 20B	nucleus; cytoplasm; microtubule; microtubule associated complex	1439695_a_at

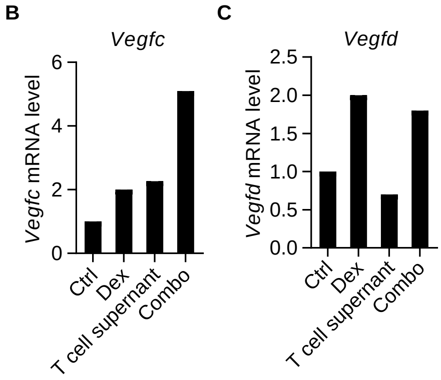

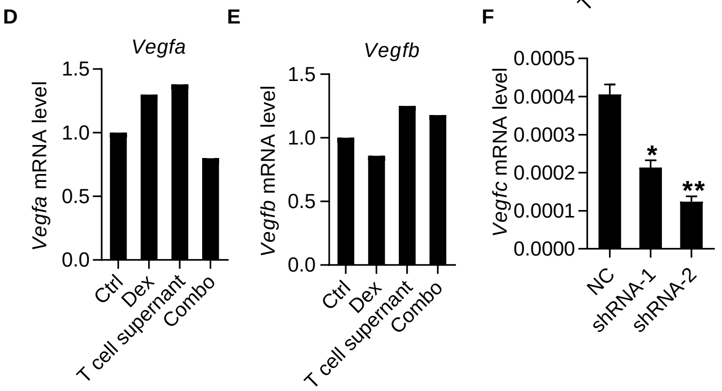

Figure S2. Expressions of VEGF family members in MSCs upon Dex treatment. A) Top 9 up-regulated genes in MSCs by Dex in the presence of activated T cells. Four groups were presented, including MSCs (G1), MSCs with Dex treatment (G2), MSCs co-cultured with $CD8^+$ T cells (G3) and MSCs co-cultured with $CD8^+$ T cells and with Dex treatment (G4). B-E) mRNA expressions of *Vegfc*, *Vegfd*, *Vegfa* and *Vegfb* in MSCs stimulated with activated T cell supernatant, with or without Dex addition. F) Efficiency of short hairpin RNA (shRNA)-mediated *Vegfc* knockdown in MSCs. NC, scramble shRNA control. Data are presented as mean ± SEM. $*p < 0.05$ and $**p < 0.01$.

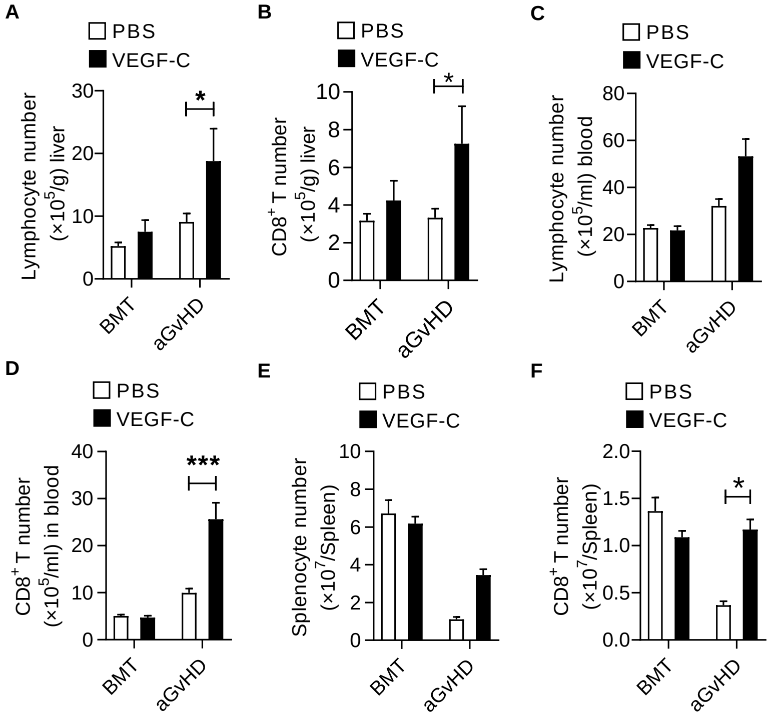

Figure S3. The influence of VEGF-C on the numbers of immune cells in the liver, blood and spleen of aGvHD mice. A-B) The numbers of lymphocytes (A) and $CD8^+$ T cells (B) in the liver of aGvHD mice treated with PBS or VEGF-C. Lethally irradiated C3H × C57 BL/6 F1 mice were adoptively transferred with bone marrow cells (5×10^6) and splenocytes (5×10^7) from C57BL/6 mice to induce aGvHD. On day 7 post aGvHD induction, mice were *i.p.* injected with VEGF-C at 10 µg per mouse daily. For the BMT group, recipient mice were adoptively transferred with bone marrow cells (5×10^6), with or without VEGF-C administration. C-D) The numbers of lymphocytes (C) and $CD8^+$ T cells (D) in peripheral blood of aGvHD mice treated with PBS or VEGF-C. E-F) Numbers of splenocytes (E) and $CD8^+$ T cells (F) in the spleen of aGvHD mice treated with PBS or VEGF-C. Data are presented as mean ± SEM. *$p < 0.05$ and ***$p < 0.001$.

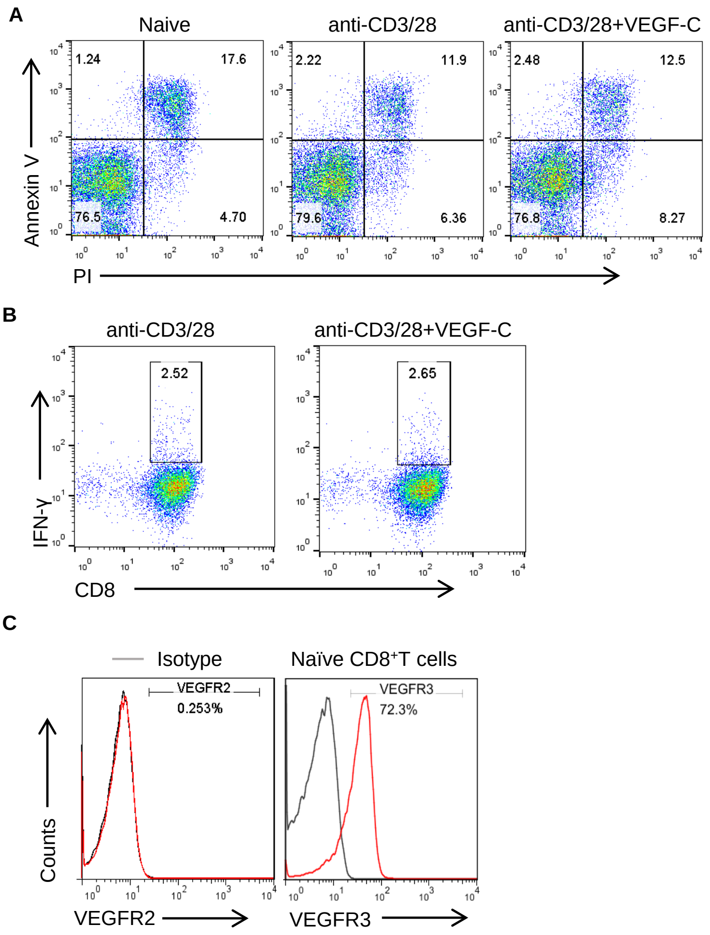

Figure S4. The effects of VEGF-C on CD8$^+$ T cell apoptosis and IFN-γ expression. A) Flow cytometric analysis of apoptotic cells among CD8$^+$ T cells activated with anti-CD3 and anti-CD28 for 48 hours, in the presence or absence of VEGF-C (800 ng/ml). B) Flow cytometric analysis of IFN-γ expression in CD8$^+$ T cells stimulated with anti-CD3 and anti-CD28 in the presence of VEGF-C. C) Flow cytometric analysis of the expressions of VEGFR2 and VEGFR3 on naïve CD8$^+$ T cells.

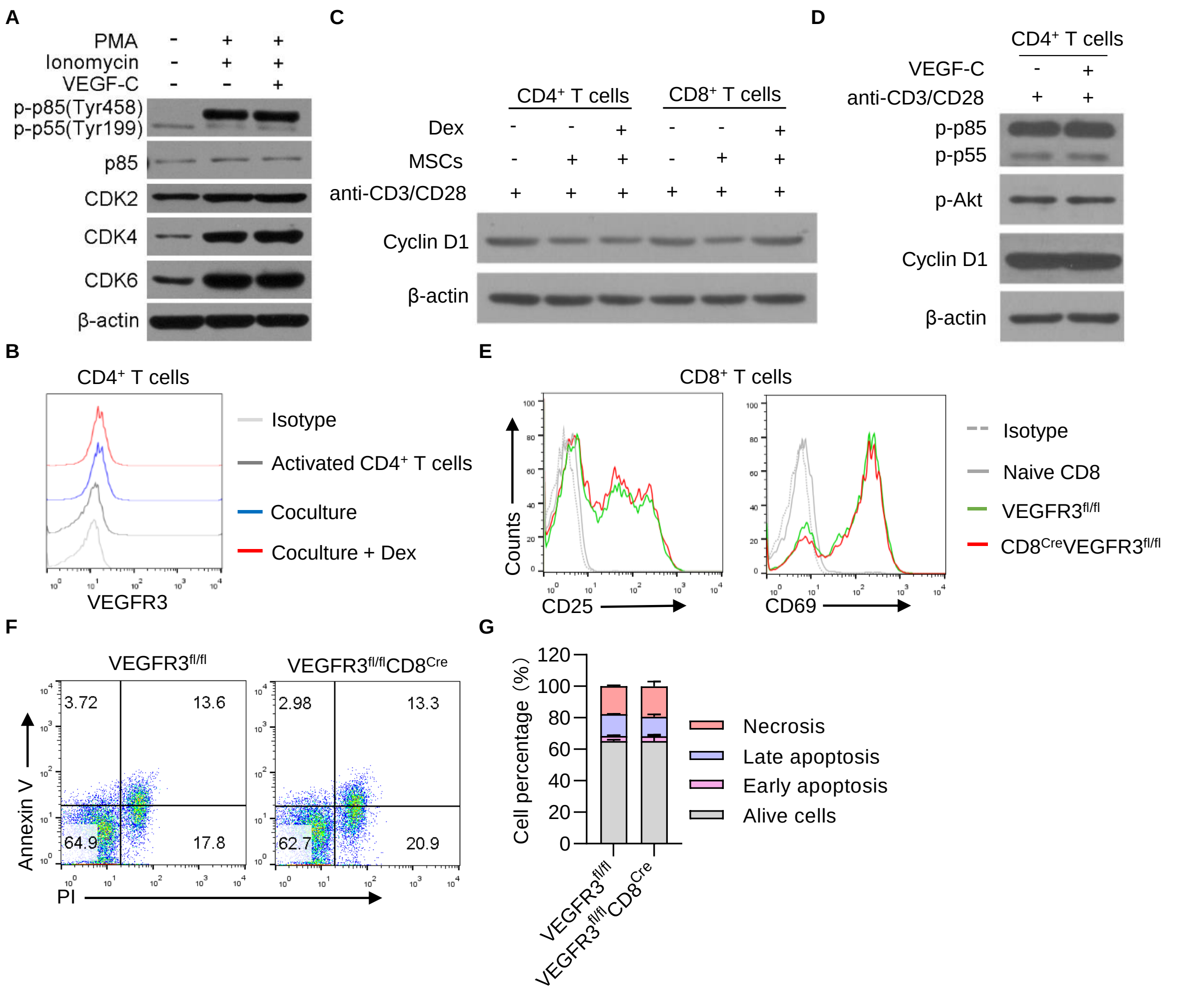

Figure S5. Activation and apoptosis in CD8$^+$ T cells isolated from VEGFR3$^{fl/fl}$ and VEGFR3$^{fl/fl}$CD8^{Cre} mice. A) Immunoblotting analysis of total p85, phosphorylated p85/p55, CDK2, CDK4, and CDK6 in CD8$^+$ T cells stimulated with PMA and ionomycin. β-actin was used as a loading control. B) Flow cytometric analysis of the expression of VEGFR3 on CD4$^+$ T cells co-cultured with MSCs, with or without Dex (10 ng/ml) additon. C) Immunoblotting analysis of Cyclin D1 expression in CD4$^+$ T cells and CD8$^+$ T cells co-cultured with MSCs, with or without Dex (10 ng/ml) addition. D) Immunoblotting analysis of the expressions of p-p85/p-p55, p-Akt and Cyclin D1 in CD4$^+$ T cells stimulated with anti-CD3 and anti-CD28 and in the presence or absence of VEGF-C (800 ng/ml). E) Flow cytometric analysis of the expressions of CD25 and CD69 on CD8$^+$ T cells activated by anti-CD3 and anti-CD28. F-G) Flow cytometric analysis of apoptotic cells in anti-CD3 and anti-CD28 activated CD8$^+$ T cells isolated from VEGFR3$^{fl/fl}$ and VEGFR3$^{fl/fl}$CD8^{Cre} mice.

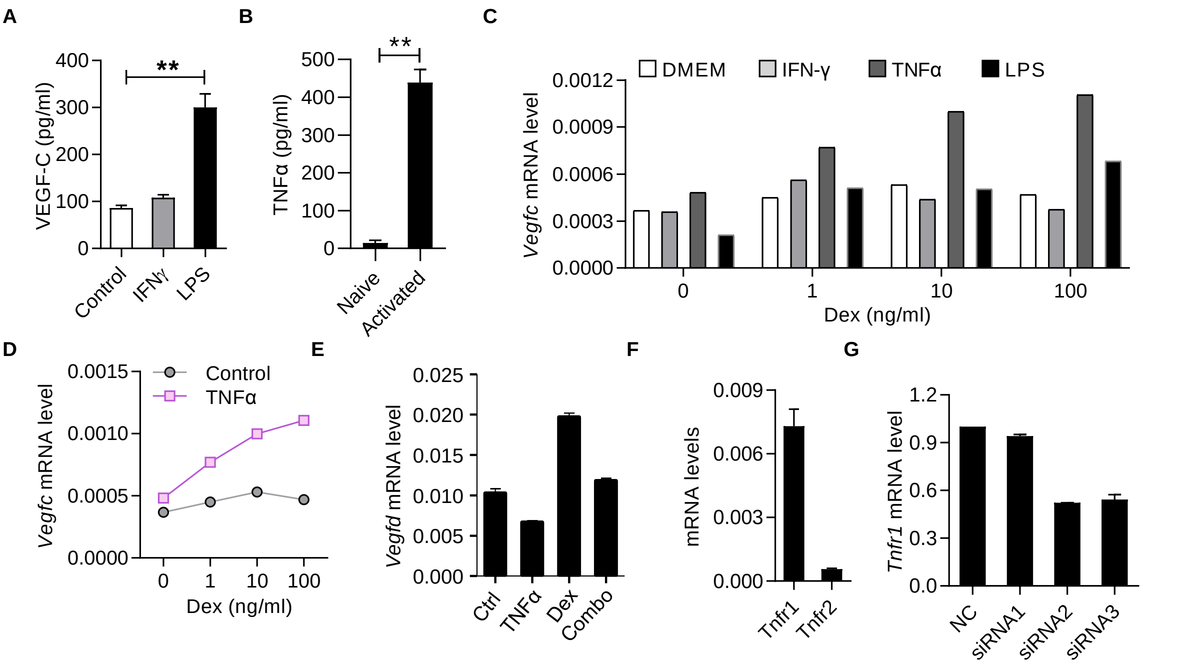

Figure S6. TNFα and Dex synergistically enhance VEGF-C expression in MSCs. A) The protein levels of VEGF-C in the supernatant of MSCs treated with PBS, LPS (100 ng/ml) or IFN-γ (10 ng/ml). B) The protein level of TNFα in the supernatant of $CD8^+$ T cells stimulated with anti-CD3 and anti-CD28. C) *Vegfc* mRNA expressions in MSCs stimulated with LPS (20 ng/ml), IFN-γ (5 ng/ml) or TNFα (5 ng/ml) and treated with Dex at indicated concentrations. D) mRNA expressions of *Vegfc* in MSCs treated with TNFα (5 ng/ml) and Dex at indicated concentrations. E) mRNA expressions of *Vegfd* in MSCs treated with TNFα (10 ng/ml), Dex (10 ng/ml) or their combination. F) mRNA expressions of *Tnfr1* and *Tnfr2* in MSCs. G) Efficiency of siRNA-mediated *Tnfr1* knockdown in MSCs. NC: scramble siRNA control. Data are presented as mean ± SEM. $^{**}p < 0.01$.
